# O.1.1-9 Clinical symptoms and body composition correlates with cardiorespiratory fitness in adults with bipolar disorder: FINEXT-BD study

**DOI:** 10.1093/eurpub/ckad133.085

**Published:** 2023-09-11

**Authors:** Ilargi Gorostegi-Anduaga, José Etxaniz-Oses, Mikel Tous-Espelosin, Sara Maldonado-Martin, Purificación Lopez, Ana Gonzalez-Pinto

**Affiliations:** GIzartea, Kirola eta Ariketa Fisikoa Ikerkuntza Taldea (GIKAFIT), Society, Sports, and Physical Exercise Research Group, Department of Physical Education and Sport, Faculty of Education and Sport-Physical Activity and Sport Sciences Section, University of the Basque Country (UPV/EHU), Vitoria-Gasteiz, Araba/Álava, Basque Country, Spain; Bioaraba Health Research Institute, Physical Activity, Exercise, and Health Group, Vitoria-Gasteiz, Basque Country, Spain; Department of Physical Education and Sport, Faculty of Education and Sport-Physical Activity and Sport Sciences Section, University of the Basque Country (UPV/EHU), Vitoria-Gasteiz, Araba/Álava, Basque Country, Spain; GIzartea, Kirola eta Ariketa Fisikoa Ikerkuntza Taldea (GIKAFIT), Society, Sports, and Physical Exercise Research Group, Department of Physical Education and Sport, Faculty of Education and Sport-Physical Activity and Sport Sciences Section, University of the Basque Country (UPV/EHU), Vitoria-Gasteiz, Araba/Álava, Basque Country, Spain; Bioaraba Health Research Institute, Physical Activity, Exercise, and Health Group, Vitoria-Gasteiz, Basque Country, Spain; GIzartea, Kirola eta Ariketa Fisikoa Ikerkuntza Taldea (GIKAFIT), Society, Sports, and Physical Exercise Research Group, Department of Physical Education and Sport, Faculty of Education and Sport-Physical Activity and Sport Sciences Section, University of the Basque Country (UPV/EHU), Vitoria-Gasteiz, Araba/Álava, Basque Country, Spain; Bioaraba Health Research Institute, Physical Activity, Exercise, and Health Group, Vitoria-Gasteiz, Basque Country, Spain; Department of Psychiatry, Bioaraba Health Research Institute, Osakidetza Basque Health Service, Vitoria-Gasteiz, Spain; CIBERSAM, Centro de Investigación Biomédica en Red Salud Mental; ilargi.gorostegi@ehu.eus; Department of Psychiatry, Bioaraba Health Research Institute, Osakidetza Basque Health Service, Vitoria-Gasteiz, Spain; CIBERSAM, Centro de Investigación Biomédica en Red Salud Mental; ilargi.gorostegi@ehu.eus

## Abstract

**Purpose:**

Bipolar disorder (BD) is a mental illness involving mood disorder, characterized by alternating normal mood, manic and depressive symptom. People with BD are associated with an unhealthy lifestyle. Assessment of cardiorespiratory fitness (CRF) indicates the overall health and can be directly measured and assessed by the cardiopulmonary exercise test and peak oxygen uptake (VO2peak), respectively. The CRF in the BD population may be altered due to sex, age, depression symptoms, and inactivity. The aim of this study was to analyze possible correlations of CRF with clinical symptoms and body composition variables.

**Methods:**

Participants (n = 53, 44.9 ± 13.2 yr old) with BD were assessed with (1) body mass index and fat body mass (FBM) percentage; (2) upright bicycle ergometer using an incremental ramp protocol to get the VO2peak; and (3) symptoms of the disease [Hamilton Depression Rating Scale (HRSD) and Clinical Global Impressions Scale (CGI-S)]. Correlation analyses were performed among clinical symptoms, BMI, and VO2peak.

**Results:**

The studied population showed overweight based on BMI (29.4±6.8 kg/m2), and obesity taking into account the fat mass percentage (33.6±10.6 %). According to Kaminsky et al., participants were classified in the 40th percentile with a low physical condition (VO2peak =25.8±8.4 mL·kg-1·min-1). Spearman's rho correlation coefficients demonstrated an inverse relationship between VO2peak (mL·kg−1·min−1) and HRSD (B=-0.286; P = 0.040); VO2peak (mL·kg−1·min−1) and CGI-S (B=-0.277; P = 0.047); and VO2peak (mL·kg−1·min−1) and FBM (B=-0.606; P < 0.001).

**Conclusions:**

In population with BD a significantly higher CRF was detected in those with lower clinical symptoms, as well as lower FBM. Exercise interventions for improving CRF should be promoted in this population to better control clinical symptoms.

